# Age‐Specific Normative Values for the Sitting‐Rising Test in Brazilian Children and Adolescents

**DOI:** 10.1111/jpc.70507

**Published:** 2026-07-17

**Authors:** Cíntia de Oliveira Cunha, Ana Luiza Righetto Greco, Gustavo Henrique Leite de Souza, Layra Alves Guimarães, Thailyne Bizinotto, Celmo Celeno Porto, Cibelle Kayenne Martins Roberto Formiga

**Affiliations:** ^1^ State University of Goiás Goiânia Brazil; ^2^ Federal University of Goiás Goiânia Brazil

**Keywords:** adolescents, body composition, child, motor skil, SRT

## Abstract

**Introduction:**

Functional assessment of children and adolescents is essential for monitoring motor development and for the prevention of deficits that may impact health and quality of life. In primary care contexts, the implementation of simple and cost‐effective tests has been a pragmatic strategy, particularly in low‐ and middle‐income countries. The sitting‐rising test is noteworthy for its clinical and community relevance, although normative values for paediatric populations remain unavailable.

**Objective:**

To establish reference values for the Sitting‐rising Test in children and adolescents aged 5–14 years, to investigate the influence of body mass index on functional performance, and to compare results between sexes.

**Methods:**

A cross‐sectional study was conducted with 596 schoolchildren (aged 5–14 years) assessed using the Sitting‐rising Test and anthropometric measures. Reference values were defined by age‐specific percentiles. The association between body mass index and performance was analysed using linear regression, and sex differences were tested with the chi‐square test.

**Results:**

Sitting‐rising Test performance was classified into three categories: below expected (≤ P10), adequate (> P10 and ≤ P90) and above expected (> P90). Body mass index accounted for 17.4% of the performance variation, indicating that greater body composition was associated with poorer test results. No significant sex differences were observed.

**Conclusion:**

This study provides, for the first time, normative Sitting‐rising Test values for children and adolescents aged 5–14 years. The findings highlight the SRT as a simple, low‐cost and effective tool for early detection of functional deficits, especially in resource‐limited settings.

## Summary

- Sitting‐rising Test is a practical, inexpensive and functionally relevant tool for motor assessment
- Excess weight increases the load on the lower limbs and restricts movements such as sitting and standing up
- This study is the first to establish normative SRT values for children and adolescents aged 5–14 years.
- The SRT should be included in clinical, school and primary care settings, and in policies promoting healthy, active lifestyles among youth.

## 1 Introduction

Human motor development is a continuous process influenced by interactions among biological, environmental and social factors such as body composition, growth, nutrition, lifestyle and socioeconomic conditions [1, 2, 3].

Assessing motor development enables the early identification of alterations, allowing timely interventions and contributing to better health outcomes. In children and adolescents, the combined evaluation of motor development and body composition supports healthy growth and guides preventive strategies. However, increased exposure to digital media and internet use has reduced the time devoted to physical activity and outdoor play, encouraging sedentary behaviours that may negatively affect motor development. This issue is particularly relevant in Brazil, which is among the countries with the highest levels of social media use worldwide [2, 4, 5].

Monitoring motor development in schools and primary health care settings is essential for the early detection of functional deficits. Physicians, physiotherapists, physical education teachers and other health professionals can identify below‐expected performance during daily activities, contributing to the prevention of future neuromuscular impairments and the promotion of healthy lifestyle habits throughout life. In this context, widely used functional tests assessing strength, muscular endurance, balance, flexibility and agility, combined with anthropometric indicators such as body mass index (BMI), constitute valuable tools for monitoring health status and motor development in healthy paediatric populations [2, 6, 7, 8].

Among the available instruments, the Sitting‐rising Test (SRT) stands out for assessing motor sequence typical of daily activities, involving the participants' transition from standing to sitting on the floor and returning to an upright position. While it has been extensively validated in adults and older adults, showing associations with functional capacity and fall risk [9], its use in children and adolescents remains limited.

In school‐aged populations, comprehensive batteries, such as Eurofit, FitnessGram and Alpha‐fit, are typically used for functional assessment; however, these tools, though rigorous and validated, require increased time, infrastructure and financial resources, which may restrict their applicability in resource‐limited school or community contexts [8, 9, 10].

In this context, the SRT is a simple, low‐cost and functional tool that can be integrated into school and primary care programmes, especially in low‐ and middle‐income countries, to identify motor impairments and guide cost‐effective interventions [11, 12]. Nonetheless, the establishment of reference values is essential to support clinical, educational and scientific interpretations of performance across different ages.

Although previous research has explored associations between BMI and SRT performance in Brazilian schoolchildren [13], normative values stratified by age and sex remain unavailable for paediatric populations. This gap limits the use of the SRT as a screening instrument in childhood and school‐based settings.

Therefore, the present study aimed to establish reference values for functional performance in the SRT in Brazilian children and adolescents aged 5–14 years, to analyse the influence of BMI on functional performance, and to compare the results between sexes.

## 2 Methods

### 2.1 Study Design

This was an observational, cross‐sectional study with a quantitative and descriptive approach.

### 2.2 Population

The convenience sample consisted of 596 children and adolescents (311 boys) recruited from public schools in Goiânia. Ten schools were invited to participate; however, only four agreed to participate in the study. Data collection took place between 2019 and 2023. Eligible participants were boys and girls aged 5–14 years, regularly enrolled in school, able to understand simple instructions, whose parents and/or guardians provided informed consent and who themselves expressed willingness to participate.

Exclusion criteria included parental report of musculoskeletal disorders, fractures of the lower limbs or neurological conditions such as Down syndrome, muscular dystrophies, cerebral palsy, hydrocephalus and myelomeningocele.

The sample size calculation was performed using the G*Power software (version 3.1.9.2), considering an effect size (*f*) = 0.25, significance level (*α*) = 0.05 and statistical power = 0.95. To account for the multicentre design of the study, a design effect (DEFF = 2.92) was applied, assuming an average cluster size of 25 participants and an intraclass correlation coefficient (ICC) = 0.08, in addition to a 20% increase to compensate for potential sample losses.

The study was approved by the Research Ethics Committee of the State University of Goiás (CEP/UEG) (protocol no. 51565821.9.3001.8113) and conducted in accordance with the Brazilian Guidelines and Norms for Research Involving Human Subjects (Resolution 466/2012, National Health Council). Parents or guardians provided written consent, and children gave assent through an age‐appropriate document.

### 2.3 Variables and Assessment Instruments

Body weight was measured using a Glass 10 G‐Tech digital scale, activated by touch. Height was measured with a portable Avanutri stadiometer, consisting of a rigid ruler with a movable pointer fixed to the base. From these measures, BMI was calculated as weight (kg) divided by height squared (m^2^). BMI classification followed the World Health Organization [4] parameters for children aged 5–4 years, using percentiles to categorise underweight, normal weight, overweight and obesity.

The SRT [12] was used to assess functional performance. Starting from a standing position, participants were instructed to sit and then rise from the floor without using their hands for support and without losing balance. The standardised command was: ‘Try to sit down and stand up without losing your balance, using the fewest supports possible’. A mat was placed behind the participant to ensure safety during the execution.

A maximum score of 5 points was assigned to each task (sitting and rising), totalling 10 points. For each support used, 1 point was subtracted, and for each episode of imbalance, 0.5 points were deducted. Two attempts were allowed for each task, with only the best performance considered.

### 2.4 Procedures

The research team conducted on‐site visits to schools and distributed invitation letters in classrooms, addressed to parents and/or guardians. The letters included information on the study's objectives and procedures, as well as the informed consent form. Along with the letters, a screening form was provided with questions regarding sex, age, presence of neurological or orthopaedic conditions, diagnoses that could compromise motor and/or cognitive functions and continuous medication use.

After obtaining written informed consent and assent, participants attended a data collection session at the school sports court. Body weight and height were measured, BMI was calculated and participants subsequently performed the SRT on a mat following standardised instructions provided by the researchers.

All procedures were carried out by two evaluators previously trained in administering the test (95% inter‐rater reliability). Each participant was allowed two attempts after the evaluator's demonstration, with only the best performance considered for analysis.

### 2.5 Data Analysis

Descriptive analyses characterised the sample by sex, weight, height, BMI and BMI classification. Continuous variables were expressed as mean ± SD, and categorical variables as frequencies. Chi‐square and linear regression tested sex differences and BMI influence on SRT performance. Age‐specific percentiles defined normative values (5th, 10th, 25th, 50th, 75th, 90th and 95th). Analyses were conducted in SPSS 23.0 with *p* < 0.05.

## 3 Results

Of the 750 children who received invitation letters, 620 returned them with the forms duly completed and parental consent. Among these, 20 children were not assessed due to absence on the data collection days, and another 4 were transferred to different schools during the study period. A total of 596 children were evaluated, 52.2% male (*n* = 311) and 47.8% female (*n* = 285), recruited from four public schools. The sample presented a mean body weight of 37.22 ± 13.25 kg, mean height of 1.41 ± 0.15 m and mean BMI of 18.10 ± 3.76 kg/m^2^.

Regarding BMI classification, 76% of the participants were considered eutrophic. Table 1 presents the characterisation of the participants according to each age group.

**TABLE 1:** Characterisation of participants by sex, body weight, height and BMI (*n* = 596).

Age (year)	Sex (female: male)	Weight (kg)	Height (m)	BMI (kg/m^2^)	Classification (%) (normal weight: overweight)
5	10:10	20.50 ± 4.24	1.14 ± 0.05	15.5 ± 2.26	80.00:20.00
6	17:10	22.58 ± 4.08	1.19 ± 0.05	15.69 ± 2.25	85.20:14.80
7	29:36	26.76 ± 6.79	1.24 ± 0.06	16.58 ± 2.93	76.90:23.00
8	54:37	30.97 ± 8.22	1.31 ± 0.06	17.79 ± 3.61	68.10:31.90
9	48:52	33.66 ± 9.89	1.37 ± 0.07	17.45 ± 3.70	81.00:19.00
10	47:34	38.09 ± 10.78	1.43 ± 0.09	18.16 ± 3.43	71.60:28.40
11	19:43	43.45 ± 14.41	1.49 ± 0./06	19.12 ± 4.74	71.00:29.00
12	24:42	46.87 ± 9.48	1.54 ± 0.07	19.53 ± 3.28	74.20:25.80
13	20:26	52.53 ± 14.72	1.60 ± 0.08	20.07 ± 4.44	80.50:19.50
14	17:21	52.46 ± 9.10	1.63 ± 0.08	19.49 ± 2.67	86.80:13.20
Total	285:311	—	—	—	76.00:23.50

Abbreviations: %: percentage; BMI: body mass index; kg/m^2^: kilogram per square meter; kg: kilogram; m: meter.

### 3.1 Functional Performance

Table 2 presents the percentile values of the SRT according to age. The median value (P50) was 10 points for ages 5, 6 and 14 years; 9.5 points between 7 and 12 years; and 8.5 points at 13 years.

**TABLE 2:** Distribution of Sit‐to‐Rise Test (SRT) scores in percentiles according to age among the children and adolescents in the study sample.

Age	Percentiles (%)						
	5	10	25	50	75	90	95
5	3650	6500	8375	10 000	10 000	10 000	10 000
6	5700	7200	9000	10 000	10 000	10 000	10 000
7	7500	8000	9000	9500	10 000	10 000	10 000
8	7100	7500	9000	9500	10 000	10 000	10 000
9	6025	7050	9500	9500	10 000	10 000	10 000
10	6000	7500	9000	9500	10 000	10 000	10 000
11	5575	6650	8000	9500	10 000	10 000	10 000
12	5350	6500	8000	9500	10 000	10 000	10 000
13	5500	6350	7500	8500	9500	10 000	10 000
14	6475	7900	8875	10 000	10 000	10 000	10 000

Functional performance in the SRT was categorised as follows: (1) below expected, when the score was equal to or below the 10th percentile; (2) adequate, when the score was above the 10th percentile and equal to or below the 90th percentile; and (3) above expected, when the score was above the 90th percentile.

Table 3 presents the influence of body composition, represented by Body Mass Index (BMI), on the functional performance of the schoolchildren evaluated.

**TABLE 3:** Influence of BMI on SRT.

IMC	*R* ^2^	*B*	IC 95%	*p*
Girls	0.170	−0.153	(−0.19)–(−0.11)	< 0.001
Boys	0.180	−0.139	(−0.17)–(0.10)	< 0.001
Total	0.174	−0.145	(−0.17)–(−0.12)	< 0.001

Abbreviations: B: regression coefficient; BMI: body mass index; CI: confidence interval; *R* ^2^: coefficient of determination.

BMI explained 17.4% of the variance in TSL scores (*R* ^2^ = 0.174; *p* < 0.001), establishing itself as a significant predictor of functional performance. For each one‐unit increase in BMI, there was an average decrease of 0.145 points in TSL scores, indicating that schoolchildren with higher BMI tend to present lower functional performance.

In the sex‐stratified analysis, among girls, BMI explained 17.0% of the variance in TSL (*R* ^2^ = 0.170; *p* < 0.001; *B* = −0.153; 95% CI: −0.19 to −0.11), demonstrating a significant negative association. Thus, each one‐unit increase in BMI resulted in an average decrease of 0.153 points in functional performance. Among boys, BMI explained 18.0% of the variance in TSL scores (*R* ^2^ = 0.180; *p* < 0.001; B = −0.139; 95% CI: −0.17 to −0.10), also indicating a significant decline in functional performance with increasing BMI.

Table 4 presents the comparison between sexes. No statistically significant differences were observed across most age groups (*p* > 0.05), suggesting similar performance between boys and girls, except at age 14, when girls exhibited superior performance.

**TABLE 4:** Comparison of performance between girls and boys in the total sample and stratified by age.

Age (years)	Boys		Girls		*p*
	Mean	SD	Mean	SD	
5	9.4	±1.7	8.6	±2.2	0.37
6	9.3	±1.3	9.2	±1.3	0.73
7	9.5	±0.6	9.1	±1.4	0.20
8	9.2	±1.0	9.2	±1.1	0.95
9	9.2	±1.2	9.1	±1.6	0.70
10	9.2	±1.3	9.0	±1.1	0.38
11	8.7	±1.5	9.1	±1.4	0.42
12	8.8	±1.5	8.9	±1.4	0.90
13	8.3	±1.3	8.7	±1.4	0.32
14	9.0	±1.3	9.6	±0.5	0.03
Total	9.0	±1.2	9.0	±1.3	0.81

Abbreviation: SD: standard deviation.

## 4 Discussion

The present study aimed to establish reference values for the functional performance of schoolchildren aged 5–14 years in the TSL, in addition to analysing the influence of BMI on functional performance and comparing the results between sexes. The mean SRD score was 10 points at ages 5, 6 and 14 years; 9.5 points between 7 and 12 years; and a slight decrease to 8.5 points at age 13, suggesting relative stability of functional performance across age groups, with variations possibly associated with BMI. Higher BMI values were related to poorer SRD outcomes. In the sex‐based comparison, no significant differences were found in most age groups, except at age 14, when girls showed superior performance.

The age‐specific percentile distribution allowed the establishment of reference values for functional performance, facilitating the classification of children and adolescents with performance below, within or above the mean for their age group. The 10th percentile was defined as the cutoff point due to the consistent pattern of performance decline in relation to the median (P50), a criterion that proved appropriate for all ages analysed.

Our findings are consistent with previous evidence. A Brazilian study conducted in 2011, involving 6238 individuals aged 8–17 years, identified progressive improvements in strength and physical fitness tests across age groups, reinforcing that motor development is gradual throughout childhood and adolescence [14]. Our results show that SRT performance remains stable or slightly improves with age, reflecting motor maturation. Latin American studies offer comparable findings.

A Peruvian investigation published in 2021 with nearly 1800 children and adolescents aged 6–16 years assessed the reproducibility of two motor competence tests included in the *Körperkoordinationstest für Kinder* (KTK). The authors demonstrated that percentile‐based cutoff points allow classification of motor competence into low, medium and high levels [15]. This categorisation model was not applicable here, as the SRT uses a point‐deduction system for supports or balance losses.

Another point of convergence concerns the relationship between body composition and functional performance. In our sample, we identified a significant negative association between BMI and SRT scores: the higher the BMI, the lower the performance, with the index explaining approximately 18% of the variance in the results. These findings align with studies showing an inverse relationship between higher BMI and motor performance in children aged 6–13 years during running, jumping or balance tasks [15, 16].

An African study involving 1037 children aged 6–12 years from Ghana and South Africa reported that each one‐point increase in BMI raised the likelihood of poor static balance by more than 20% [17]. Our results confirm that body composition directly affects functional performance. An Italian study with over 30 000 children found sex differences in fitness, especially flexibility [18]. In contrast, we observed no major sex differences except at age 14, when girls outperformed boys in the SRT.

Such differences between studies may be explained by multiple factors, including cultural contexts, physical activity patterns, lifestyle characteristics and exposure to sedentary behaviours. Excessive screen time and reduced opportunities for physical activity and outdoor play, which are increasingly common among children and adolescents, may contribute to declines in motor competence and to the stronger influence of body composition on functional performance. From a biomechanical perspective, excess body weight increases the load imposed on the lower limbs and hinders movements such as sitting and rising, thereby helping to explain the association between higher BMI and poorer SRT performance. Additionally, greater body mass requires increased muscular strength, power and motor control to perform basic functional movements efficiently [19, 20, 21, 22, 23].

When comparing sexes, our findings revealed no significant differences in functional performance across most age groups assessed, except at 14 years of age, when girls demonstrated superior performance. Although boys often perform better in tasks related to muscular strength and girls tend to excel in activities involving flexibility and balance, these differences are generally subtle during childhood and early adolescence, a period characterised by ongoing biological maturation. Moreover, SRT performance depends on the integration of multiple physical capacities, including strength, flexibility, balance and motor control, which may minimise sex‐related differences. The superior performance observed among 14‐year‐old girls may be associated with earlier pubertal maturation, favouring body control and the execution of functional movements.

This study presents some limitations that should be acknowledged, including a regional sample that may limit generalisability and the lack of control for factors like physical activity, screen time and socioeconomic status. Despite its limitations, this study provides new SRT normative values for children aged 5–14 years, offering a practical, low‐cost tool for functional monitoring. The test can aid early detection of motor deficits and guide timely interventions, especially in sedentary or overweight populations.

Future studies should adopt longitudinal designs to track changes in performance over time and consider factors such as physical activity level, exposure to digital media and socioeconomic conditions in order to broaden the understanding of the determinants of functional performance in the SRT.

## 5 Conclusion

This study provides, for the first time, normative values of the Sit‐to‐Stand Test (SRT) for children and adolescents aged 5–14 years, classifying performance as below expected (≤ P10), adequate (> P10 and ≤ P90) or above expected (> P90). In addition, higher BMI values were found to be associated with poorer functional outcomes, highlighting the importance of monitoring nutritional status in this age group.

The proposed percentiles provide objective reference values for interpreting the SRT, aiding early identification of motor deficits and supporting health promotion programmes. The findings highlight the SRT's value as a simple, low‐cost tool for functional screening, especially in resource‐limited settings.

It is recommended that the SRT be incorporated into clinical, school and primary care practices, as well as public policies that encourage healthy habits and active lifestyles among children, adolescents and their families, in order to prevent sedentary behaviour, overweight and impairments in motor development.

## Author Contributions

Study design: Cíntia de Oliveira Cunha, Thailyne Bizinotto, Celmo Celeno Porto, Cibelle Kayenne Martins Roberto Formiga. Data collection: Cíntia de Oliveira Cunha, Gustavo Henrique Leite de Souza, Thailyne Bizinotto, Layra Alves Guimarães, Cibelle Kayenne Martins Roberto Formiga. Data analysis: Cíntia de Oliveira Cunha, Ana Luisa Rigetto Greco, Gustavo Henrique Leite de Souza, Celmo Celeno Porto, Cibelle Kayenne Martins Roberto Formiga. Manuscript writing: Cíntia de Oliveira Cunha, Ana Luisa Rigetto Greco, Layra Alves Guimarães, Cibelle Kayenne Martins Roberto Formiga. Manuscript revision: Ana Luisa Rigetto Greco, Thailyne Bizinotto, Celmo Celeno Porto, Cibelle Kayenne Martins Roberto Formiga. Study supervision: Celmo Celeno Porto, Cibelle Kayenne Martins Roberto Formiga.

## Funding

The authors have nothing to report.

## Ethics Statement

The authors declare the study was approved by the Research Ethics Committee of the State University of Goiás (CEP/UEG) and conducted in accordance with the Brazilian Guidelines and Norms for Research Involving Human Subjects (Resolution 466/2012, National Health Council).

## Conflicts of Interest

The authors declare no conflicts of interest.

## Data Availability

The data that support the findings of this study are available on request from the corresponding author. The data are not publicly available due to privacy or ethical restrictions.
